# Concurrent sigmoid volvulus and caecal adenocarcinoma in a paraplegic patient with neurogenic bowel: a case report

**DOI:** 10.1093/jscr/rjag761

**Published:** 2026-08-31

**Authors:** Nakul Ganju, Alvin Billey, Juan Carlos Santiago-Gonzalez, Angesom Kibreab

**Affiliations:** Department of Surgery, Howard University Hospital, 2041 Georgia Avenue NW, Washington, DC 20060, United States; Department of Surgery, Howard University Hospital, 2041 Georgia Avenue NW, Washington, DC 20060, United States; Department of Surgery, Howard University Hospital, 2041 Georgia Avenue NW, Washington, DC 20060, United States; Department of Surgery, Howard University Hospital, 2041 Georgia Avenue NW, Washington, DC 20060, United States

**Keywords:** sigmoid volvulus, neurogenic bowel, paraplegia, colorectal cancer, caecal adenocarcinoma, perioperative management

## Abstract

A 76-year-old man with paraplegia and neurogenic bowel dysfunction presented with progressive abdominal distension and altered bowel habits. Contrast-enhanced computed tomography demonstrated sigmoid volvulus and an incidental 5 × 4 cm ileocaecal mass. After endoscopic detorsion, multidisciplinary review recommended definitive resection during the same admission. He underwent right hemicolectomy with ileocolic anastomosis and sigmoidectomy with colorectal anastomosis. Histopathology confirmed moderately differentiated caecal adenocarcinoma (pT3N1a, R0). The postoperative course was complicated by aspiration pneumonitis and femoral deep venous thrombosis, both managed successfully. This case illustrates that neurogenic bowel dysfunction may mask coexistent colorectal malignancy, and that unexplained microcytic anaemia in a patient presenting with volvulus warrants comprehensive colonic evaluation. Perioperative management in this population requires multidisciplinary input to address the elevated risks of cardiopulmonary and thromboembolic complications.

## Introduction

Sigmoid volvulus in elderly or chronically immobilized patients results from combined anatomic and functional factors: a redundant sigmoid colon with a narrow mesenteric base, chronic constipation, dysmotility, neurogenic bowel dysfunction, prior abdominal surgery, and comorbidities such as neuropsychiatric disease or diabetes [1, 2]. In patients with paraplegia, impaired autonomic innervation and structural enteric changes promote progressive colonic redundancy and stasis, predisposing to torsion [1, 3]. Computed tomography (CT) is the diagnostic investigation of choice, with sensitivity approaching 100% and specificity exceeding 90%; the characteristic whirl sign confirms torsion [1, 2]. In haemodynamically stable patients, endoscopic detorsion is the preferred initial intervention, though recurrence rates of 43%–86% support elective sigmoid colectomy during the same admission [1, 2, 4–8]. Colorectal carcinoma coexists in ~2%–5% of sigmoid volvulus evaluations, underscoring the importance of complete colonic assessment [2, 9].

## Case report

**Figure 1:**
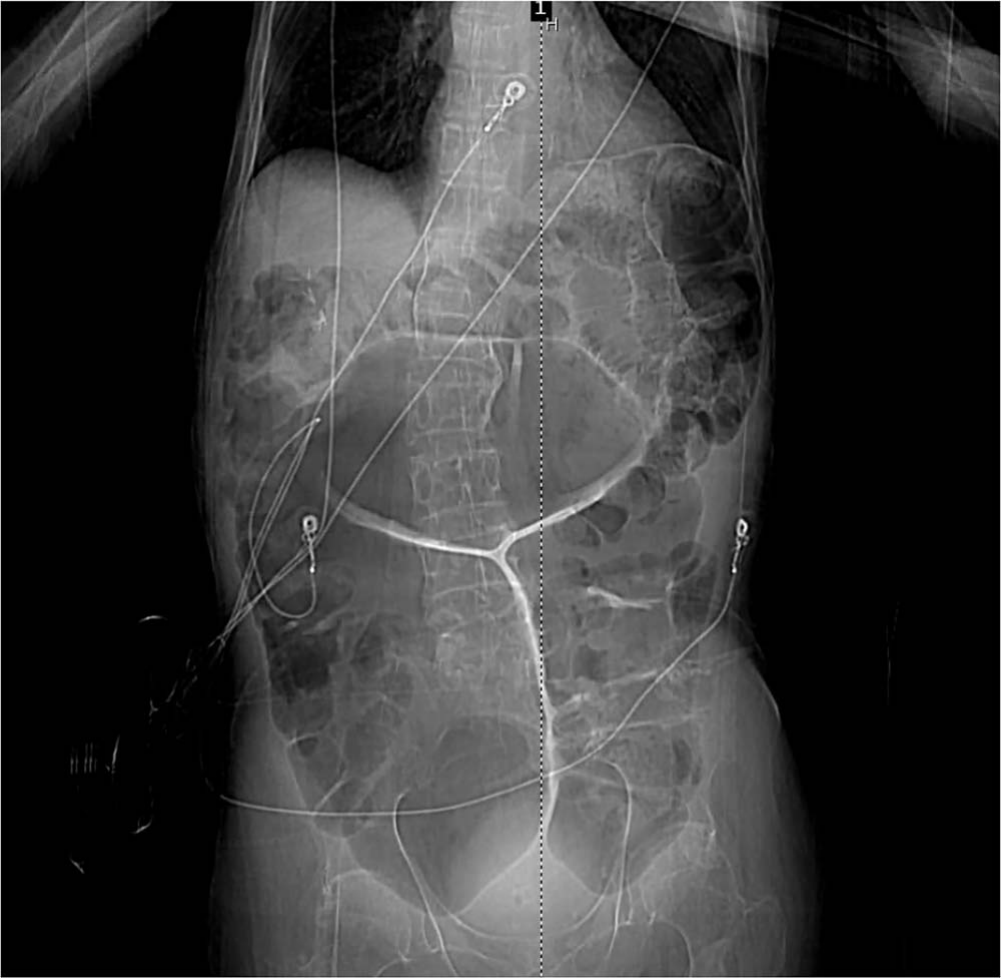
CT scout topogram of the abdomen and pelvis demonstrating marked colonic dilatation with haustral markings, consistent with large bowel obstruction.

A 76-year-old man with paraplegia, neurogenic bowel and bladder, and prior cerebrovascular accident presented with several months of progressive abdominal distension, early satiety, and reduced oral intake. On admission, the abdomen was markedly distended and tympanitic without peritoneal signs. Laboratory investigations revealed hypokalemia (1.9 mmol/L), hyponatremia (132 mmol/L), microcytic anaemia (haemoglobin 9.3 g/dL; MCV 72 fL), and mildly elevated carcinoembryonic antigen (CEA 5.4 ng/mL). Contrast-enhanced CT demonstrated a massively dilated sigmoid colon with a mesenteric whirl sign and a 5 × 4 cm soft-tissue mass at the ileocaecal valve without radiological evidence of distant metastases (Fig. 1).

**Figure 2:**
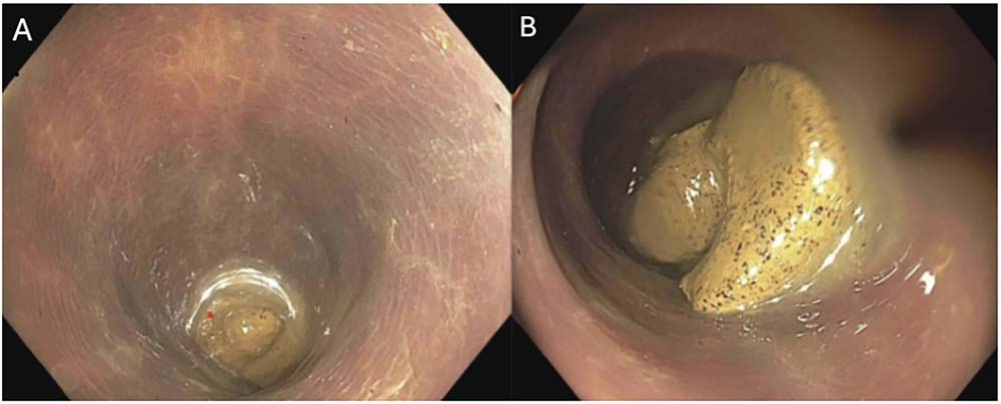
Colonoscopic views demonstrating characteristic features of sigmoid volvulus. (A) Spiral pinwheel configuration of the colonic mucosa at the point of torsion. (B) Twisted sigmoid segment with luminal obstruction.

Following electrolyte correction and fluid resuscitation, colonoscopy confirmed sigmoid volvulus with viable mucosa; endoscopic detorsion was achieved with decompression tube placement (Fig. 2). Preoperative tissue biopsy of the ileocaecal mass was not obtained: colonoscopic access was technically unstable following detorsion, perforation risk in the recently decompressed colon was considered unacceptable, and definitive surgery was independently indicated to prevent volvulus recurrence. Histological confirmation would not have altered the operative approach. Following multidisciplinary discussion and nutritional optimization, the patient underwent right hemicolectomy with ileocolic anastomosis, sigmoidectomy with colorectal anastomosis, and Stamm gastrostomy.

Histopathology demonstrated moderately differentiated caecal adenocarcinoma invading pericolic fat (pT3), with one of twenty-two lymph nodes positive (pN1a), clear margins (R0), lymphovascular invasion present, and perineural invasion absent. Adjuvant capecitabine-based chemotherapy was recommended following oncology review; regimen selection reflected frailty and comorbidity burden.

Postoperatively, the patient developed aspiration pneumonitis requiring intubation and vasopressor support, and right femoral deep venous thrombosis managed with low-molecular-weight heparin. He was successfully extubated and discharged to long-term care. Several weeks later he was readmitted with melena and haemodynamic instability while receiving apixaban (haemoglobin 6.3 g/dL). Endoscopic evaluation was deferred following palliative care discussion, in view of frailty, clinical stabilization with conservative management, and the agreed ceiling of care.

## Discussion

Neurogenic bowel dysfunction and chronic dysmotility promote sigmoid volvulus through progressive colonic redundancy, impaired motility, and faecal stasis [1–3]. Without definitive surgery, recurrence and mortality remain high; early resection is therefore preferred in suitable patients [4–8].

Coexistent colorectal malignancy in the setting of volvulus has been reported, though most published cases involve tumours within the sigmoid colon itself [9]. This case is notable for a right-sided caecal adenocarcinoma anatomically distinct from the torsion site, emphasizing that malignant pathology may coexist independently and requires proactive exclusion. In neurologically impaired patients, baseline dysmotility may mask constitutional symptoms of malignancy; microcytic anaemia in this context should prompt comprehensive colonic evaluation beyond simple detorsion.

The decision to proceed to oncological resection without preoperative biopsy is justified when histology would not alter operative management, when technical access is limited, and when perforation risk is significant [2, 10]. Multidisciplinary perioperative optimization is essential in paraplegic patients, who face elevated risks of thromboembolism, aspiration, and poor wound healing [2, 9, 11]. This case underscores that dual pathology in a high-risk host demands coordinated surgical, oncological, and palliative care input from the outset.
